# Assessing the Impact of Parental Depressive Symptoms on Offspring Temperament and Development in Infancy

**DOI:** 10.4172/2167-1044.S1-005

**Published:** 2014-05-01

**Authors:** Nancy Huynh, Jackie Finik, Jenny Ly, Yoko Nomura

**Affiliations:** 1 Macaulay Honors College, CUNY, New York, New York, USA; 2 Department of Psychology, Queens College, CUNY, Flushing, New York, USA; 3 Division of Child and Adolescent Psychiatry, Department of Psychiatry, Icahn School of Medicine at Mount Sinai, USA

**Keywords:** Antenatal depression, Temperament, Prospective study

## Abstract

The study prospectively followed 135 women during their pregnancy and their offspring till 6 months of age, to examine the roles of maternal and paternal depression during pregnancy on offspring neurobehavioral development as measured by their early temperament. Maternal and paternal depression statuses were ascertained during the third trimester, and infant temperament was evaluated at 6 months, via mothers self-report. Multivariable general linear model was used to assess 1) the main effects of maternal and paternal depression on infant temperament and 2) the interaction effect between maternal and paternal depression on infant temperament. Results show that maternal depression, but not paternal depression, was directly associated with greater neurobehavioral impairment in offspring as evident by more difficult temperament, including lower Smiling and Laughter (p= .006), lower Soothability (p= .02), elevated Sadness (p= .04) and lower Vocal Reactivity (p= .001). Moreover, only in the presence of maternal depression, was paternal depression significantly associated with signs of offspring neurobehavioral impairment, including lower Smiling and Laughter (p= .01) lower High Pleasure Seeking (p= .03), lower Soothability (p= .05), lower Cuddliness (p= .05) and lower Vocal Reactivity (p< .0001). These findings suggest that maternal, but not paternal, depression was directly associated with infant neurobehavioral impairment. Significant interaction effect suggests that in the presence of maternal depression, paternal depression amplifies its negative valence on infant neurobehavioral development. Providing intervention services not only for depressed mothers but also their partners during pregnancy may prove to be an effective prevention strategy for suboptimal neurobehavioral development in offspring.

## Introduction

Despite the common belief that pregnancy is a happy time in a woman’s life, occurrence of depression during pregnancy reportedly range from 14% to 23% among 25 to 44 year olds [1]. Women of certain groups are at greater risk of suffering from depression during pregnancy including minorities [2], those with a past personal and family history of depression, poorer overall health, smoking, alcohol and drug abuse, unemployment, being single, uninsured, lower educational attainment, and lower socioeconomic status [3,4]. Even though there is a relatively high prevalence of depression during pregnancy among expectant mothers, depression screening at obstetric (OB) clinics is not a routine practice, resulting in missed cases of depression that deserve attention and treatment. Furthermore, the rate of detection of depression for pregnant women is reported to be extremely low (i.e., 0.8%) [4]. Under-diagnosis of depression may be, in part, due to the overlapping symptoms of depression and normal pregnancy, including sleep disturbances, weight gain, appetite disturbances, and fatigue. To make matters worse, 86% of pregnant women identified with significant depression in OB settings are more likely to have poor attendance for their prenatal appointments and avoid seeking treatment for depression [4].

Identification and treatment of maternal depression during pregnancy is especially important because adverse consequences affect both the mother and her offspring (fetus). Specifically, depression can interfere with the mother’s ability to care for herself during pregnancy, as she may be less likely to eat, sleep and follow medical recommendations from her doctor, resulting in low pregravida maternal weight [5]. Accumulating evidence shows that prenatal depression in mothers has been associated with several poor birth outcomes, such as miscarriages [6], preterm delivery [7], and fetal growth effects such as low birth weight and growth retardation [8]. Fetuses of depressed and anxious mothers observed during ultrasound exhibited less gross body activity and lower fetal growth [9]. Depression during the second trimester of pregnancy is adversely associated with not only fetal growth changes, but also offspring temperament [10]. Field et al. for example, found that infants of depressed as compared to those of non-depressed mothers, showed elevated cortisol levels, a stress hormone secreted by the adrenal glands, and decreased levels of dopamine and serotonin [11], which were associated with a slower rate of development [12] and known to disrupt emotional regulation in infants [13]. Other studies demonstrated that maternal depression was associated with infants’ behaviors in early infancy and childhood. For example, infants of depressed mothers often exhibit lower vagal tone which has been associated with less attentiveness and fewer facial expressions, when compared to infants born to mothers who were not depressed during the neonatal period [11]. Eighteen month old infants whose mothers were depressed during the second trimester (18 to 32 weeks gestation), compared to infants of non-depressed mothers showed greater delay in development [14].

Most studies, to date, have centered on maternal prenatal depression; however, paternal depression during his partner’s pregnancy and infancy has received little attention and is poorly understood. Among those few studies, one study showed that paternal depression during his partner’s pregnancy might be a risk factor for excessive infant crying [15]. Field et al. also found that prenatal paternal and maternal depression scores were relatively similar. This is especially important because both mothers and fathers provide care for their newborn babies [16]. Infants were also found to have better interactions with their non-depressed fathers compared to their depressed mothers, which suggests that non-depressed fathers may buffer the negative effects of maternal depression on infant interaction behavior [17]. While various factors such as young age, negative mood, gender role differences [18], marital dissatisfaction, and low social support [19] predicted distress in mothers and their partners during pregnancy, it is plausible that fathers are one of the main sources of emotional support for mothers, and vice versa.

Although we have previously found that children with both parents diagnosed with Major Depressive Disorder (MDD) have a 32% increased risk for psychiatric disorders than those who have only one parent with MDD (Nomura et al.), little is known about the combined effects of both parents having depression during pregnancy and its effect on young infants. The examination of depressive symptoms of both parents during pregnancy, and those independent and interactive effects on early infant temperament, may shed light towards providing effective couple/family intervention plans during pregnancy.

## Methods

### Participants

A total of 135 pregnant women, less than 24 weeks in gestation, were recruited at the prenatal obstetrics and gynecological (OB/GYN) clinic at Mount Sinai Hospital, which serves predominantly low-income ethnic minorities from East Harlem and the South Bronx in New York City. Included participants received prenatal care or delivered at Mount Sinai Hospital and were followed from the 2nd trimester of their pregnancy to 6 months after delivery. The 2nd trimester was selected as the starting point, as the study population consisted of predominantly financially disadvantaged women, who were likely to start their prenatal care later in gestation.

Exclusion criteria for participation included HIV infection, maternal psychosis, maternal age <15 years, life-threatening medical complications of the mother, and congenital or chromosomal abnormalities of the fetus. All subjects were consented per protocol approved by the Institutional Review Boards at Icahn School of Medicine at Mount Sinai.

## Procedure

### Maternal and Paternal Depression during Pregnancy

After confirming eligibility and obtaining informed consent, demographic information, including maternal age, ethnicity, education level, welfare status, marital status, and previous obstetric histories were obtained. During the third trimester, Family History Screen (FHS) [20], a valid screening tool [21] generally administered to one informant, was used for assessing family history of disorders, based on the core symptoms of fifteen psychiatric disorders of the informant (infant’s mother) and the family member (infant’s father), including Major Depressive Disorder (MDD), bipolar disorder, panic disorder, generalized anxiety, agoraphobia, simple phobia, social phobia, obsessive-compulsive disorder, psychosis, antisocial personality disorder, separation anxiety, conduct disorder, attention-deficit hyperactive disorder, alcohol abuse/dependence, drug abuse/dependence, and suicide attempts or completed suicide. Positive responses to any of these screening questions serve as a gate for impairment, duration and/or exclusion of the disorder. In this study, positive status for MDD was used for both maternal and paternal depression.

### Infant Temperament

Approximately six months (twenty-four weeks) after the birth of their baby, participants reported their infant’s behavior and temperament using the self-report Infant Behavior Questionnaire-Revised (IBQ-R) Short [22]. The IBQ-R is a 91-items questionnaire used to assess infants’ behavior and temperament on 14 different domains. Activity Level refers to the baby’s gross motor activity. Distress to Limitations is defined as baby’s fussing, crying or showing distress. Fear describes the baby’s startle or distress to sudden changes in stimulation. Duration of Orientating is the baby’s attention to and/or interaction with a single object for extended periods of time. Smiling and Laughter is defined as smiling or laughter from the child in general caretaking and play. High and Low Pleasure items refer to the amount of pleasure or enjoyment related to stimulus characteristics. Soothability refers to baby’s reduction of distress when soothing techniques are used by caretaker. Falling Reactivity (i.e., rate of recovery from distress) is defined as rate of recovery from peak distress, excitement, or general arousal, and ease of falling asleep. The Cuddliness scale refers to the baby’s enjoyment while held by a caregiver. Perceptual Sensitivity is the amount of detection of low intensity stimuli from the external environment. Sadness refers to the general low mood and activity related to physical state, or inability to perform a desired action. Approach is excitement and positive anticipation of pleasurable activities. Lastly, Vocal Reactivity is the amount of vocalization exhibited by the baby in daily activities. The mothers were asked to report on a seven-point Likert scale the relative frequency of specified infant reactions in the past week with an option to indicate if she had not observed her baby in the situation in question. All procedures were approved by the Institutional Review Boards at Cities University of New York, Queens College and Icahn School of Medicine at Mount Sinai.

### Statistical method

First, descriptive statistics were conducted to evaluate the distribution, mean, and standard deviation (SD). The IBQ-R consists of 14 sub-scales. As those 14 subscales are inherently correlated to a certain degree, we chose to use multivariable analysis using general linear model (GLM). Prior to the analysis, each subscale was evaluated for normality by examining the univariate indices of skewness. If the assumption of univariate normal distribution was violated, transformation (i.e, log-transformation) would be applied to achieve normality. Multivariate normality was tested with Wilks’ Lambda. In the first GLM model, we tested only the main effects of maternal and paternal depression on infant temperament. This was followed by the same model with an additional interaction term between maternal and paternal depression on infant temperament. All analyses were performed first without potential confounders, and then with potential confounders for statistical adjustment. Based on our prior findings [23, 24], sex of the infant, mother’s race and marital status, were a priori determined as potential confounders.

## Results

### Demographic characteristics

Of those 135 mothers who participated in the study, depression statuses for five of the mothers were unavailable resulting in 130 cases in the analysis. There were four missing values for the depression status for fathers, two missing values for mother’s education status, and two missing values for the subscales of the infant temperament (one with Duration of Orienting, Perceptual Sensitivity and Sadness and the other with Soothability). Out of the 130 mothers who gave birth, there were five sets of twins resulting in 140 infants in the study.

Descriptive statistics for the demographic characteristics are presented in Table 1. The average age of the mothers was 27 years (SD=6). Approximately 50% of the mothers are Latina, 33% are Black, 7% are White, 6% are Asian, and 4% identified their ethnicity as other. Many of the participants are of low socioeconomic backgrounds, 33% have not completed high school, and 88% are receiving Medicaid, a health insurance program for low-income families and individuals living in the United States. 72% reported that they are single. 56% of their offspring are male and 44% are female.

### Main effects of maternal depression, paternal depression on infant temperament

Prior test of skewness found that all of the subscales were normally distributed. This was followed by the examination of the main effects of maternal and paternal depression on infant outcomes. Table 2 shows the results of infant temperaments by maternal and paternal depression without confounders (unadjusted) and with confounders (adjusted). Initial unadjusted GLM with maternal and paternal depression shows that, compared to infants of mothers with depression, infants of mothers without depression had higher scores on Smiling and Laughter (6.15 vs. 4.76, p= .006), Soothability (5.85 vs. 5.09, p= .02), and Vocal Reactivity (5.87 vs. 4.39, p= .001) and had lower scores on Sadness (2.99 vs. 3.69, p= .04). Except for Sadness, which was only marginally significant (p=.07), those associations remained significant after controlling for potential confounders. Additionally, Cuddliness (6.08 vs. 5.51, p= .07) was marginally significant after controlling for potential confounders. On the contrary, there was no notable difference in infants of fathers without depression than infants of fathers with depression in both unadjusted and adjusted (after controlling for potential confounders) GLM models.

### Interaction effects of maternal and paternal depression on infant temperament

**Figure 1:**
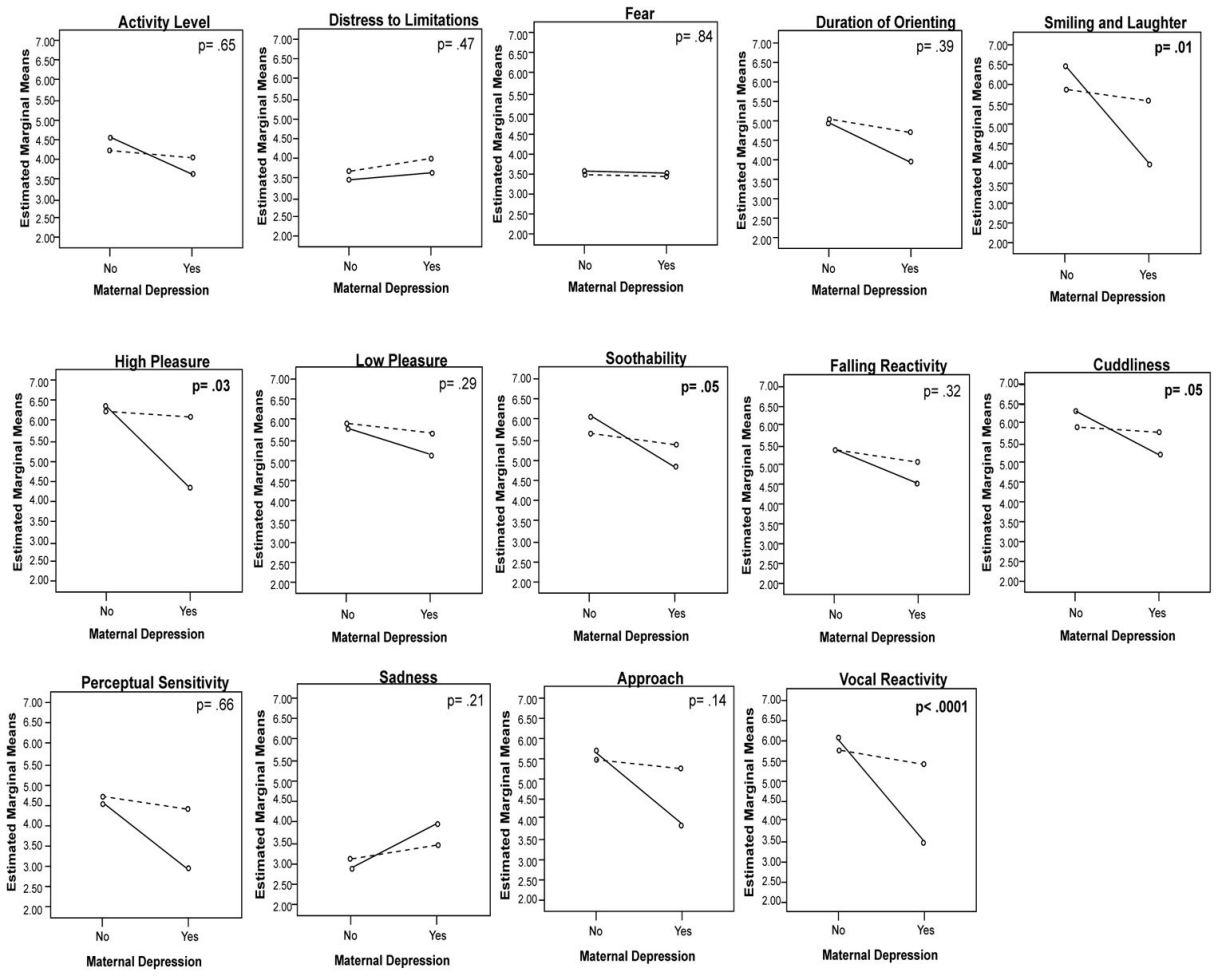
Mean plots displaying absence (no) and presence (yes) of maternal depression and paternal depression on infant temperament subscales. NB: P-value is for the interaction between maternal and paternal depression. Solid line represents the presence of paternal depression and a dotted line represents the absence of paternal depression

Figure 1 shows the presence/absence of the interaction between maternal and paternal depression on infant temperament and associated p-value for the interaction in the adjusted GLM models. Significant interaction effects were found on Smiling and Laughter, High Pleasure, Soothability, Cuddliness and Vocal Reactivity. Specifically, infants of depressed mothers did not have low scores on Smiling and Laughter unless fathers were also depressed (F1, 129 = 3.67, p= .01). Similarly, only when both the mother and the father were depressed, infants were less frequent in High Pleasure Seeking activities (F1, 129 = 3.10, p= .03), had low Soothability (F1, 129 = 2.75, p= .05), low Cuddliness when being held by a caregiver (F1, 129 = 2.67, p= .05), and low Vocal Reactivity (F1, 129= 6.80, p< .0001).

## Discussion

Our findings are consistent with existing literature which demonstrated that reactive infant temperaments and behaviors are associated with maternal depression [11,14] and extended the literature by providing the initial evidence that, while paternal depression alone was not associated with reactive temperament, there was a significant interaction between maternal and paternal depression on reactive infant temperament. The study provides two main findings: 1) maternal depression during pregnancy was directly associated with more reactive infant temperament (i.e., lower scores on Smiling and Laughter, lower Soothability, lower Cuddliness, greater Sadness, and lower Vocal Reactivity) and 2) paternal depression during his partner’s pregnancy was not directly associated with more reactive infant temperament but the interaction between maternal and paternal depression was an important predictor for infants reactive temperaments.

Our findings on the impact of maternal depression on infant temperament outcomes were consistent with prior research that showed that infants of depressed mothers during pregnancy had higher rates of disorganized [25] and insecure infant attachment [26] to mothers who were depressed during pregnancy. These findings may be linked to other areas of research have shown how maternal depression during the prenatal period affects how mothers treat their children. For example, it has been suggested that prenatally depressed mothers have increased risk of low maternal responsiveness [27] and were more dissatisfied in their interactions with their infants [28].

Our findings on the significant interaction between maternal and paternal depression on reactive temperament is notable. Specifically, when fathers were depressed, maternal depression was associated with reactive temperaments in infants as evidenced by lower Smiling and Laughter, lower High Reactivity, lower Soothability, lower Cuddliness and lower Vocal Reactivity. However, maternal depression was not associated with most of these reactive temperaments when the fathers were not depressed. These findings suggest that the negative effects of maternal depression on infant temperament may be amplified by paternal depression. That is, even though paternal depression by itself may appear to have little influence on early infant temperament, it may aggravate the negative effect of maternal depression on offspring temperament. These findings reveal that both maternal and paternal depression provide useful insight in identifying which infants have at greater risk of having reactive temperament in early childhood, which is likely to be associated with emotional and behavioral dysregulation later in childhood [11].

Theories as to understanding children’s risk in relation to maternal depression in pregnancy include the interaction between behavioral, biological, environmental and social factors as seen in Goodman and Gotlib’s study [29]. Genetic heritability among children whose mothers and their partners have a history of, or were depressed during pregnancy, increases their children’s vulnerability to depression by inheriting the parents’ characteristics, cognitive behaviors, environmental stressors and personality traits. Although it is beyond the scope of the current study, infants of depressed parents are often born with dysfunctional neuro regulatory mechanisms that interfere with emotional regulation processes and increase vulnerability to depression due to the exposure to neuroendocrine abnormalities, reduced blood flow, and poor health behaviors [30] it is possible that the association between maternal depression, paternal depression, and the high reactive infant temperaments we observed in our study may be related to the abovementioned prenatal mechanisms in depression during pregnancy. Having both paternal and maternal depression may be associated with not only negative biological (or genetic) consequences, but also with less optimal familial/social environment to which infants are exposed. Future studies should attempt to uncover some of these underlying biological and genetic mechanisms and clarify how having both maternal and paternal depression was disadvantageous to their offspring through the nature and nurture interaction. Since prior studies investigated the associations of depression during pregnancy and behavioral, emotional, and social problems in offspring during childhood retrospectively, little is known about whether temperamental dysregulations in infancy, predicted by prospectively ascertained maternal (and paternal) depression during pregnancy, could be used as early markers for subsequent behavioral, emotional, and social impairments in later childhood. As our participants age, we will be able to collect age-appropriate temperament profiles prospectively to monitor the trajectories of neurobehavioral development in children, which may further validate the utility of early reactive temperament profiles as a marker for subsequent emotional and behavioral dysregulation and impairment in childhood.

The study has several strengths. Having both maternal and paternal depression enables us to test the interaction effect between the two on infant temperament. Moreover, a longitudinal design allows us to follow our participants from the very beginning of their pregnancy, thorough their delivery, and to the first several months of their child’s life, as well as ascertain parental depression and infant temperament concurrently, which minimizes the recall bias and gives us a more complete understanding of our results. The study also uses an advanced statistical strategy, i.e., multivariable GLM, which allow us to test all of the 14 subscales of temperament simultaneously maximizing statistical power. As the 14 subscales of the temperaments were inherently correlated to some degree, it allows us to estimate the effect of the individual sub-scales and minimize Type II error. We were also able to obtain temperamental measures at such an early time during infancy, an important developmental period that is crucial in predicting future outcomes and behavior during childhood.

However, the current study also has limitations. First, we acknowledge that depression diagnostic outcomes for mothers and fathers were ascertained by a screener instrument and not by a clinical interview. It is possible that not all of our participants and their partners with MDD would meet the criteria for the DSM-IV [31] MDD diagnosis. While diagnostic outcomes ascertained by a clinical interview would have strengthened our findings [32], the FHS, nonetheless, is usually administered to one informant and its validity is best demonstrated for MDD for both parties [20]). Moreover, one of the main advantages of the FHS was to provide an easier assessment of psychiatric diagnoses, particularly for epidemiological studies. While we have mothers’ diagnostic measures based on the DSM-IV criteria from structured interviews, we do not have fathers’ diagnostic outcomes by the same means. The reliability obtained for maternal MDD assessed by the FHS and the SCID in the current sample was reasonable (Kappa=.64). As father’s depression is rarely considered in evaluating the association between parental and child transmission in current literature, we have chosen to use the FHS so that we could evaluate the effects of both mothers’ and fathers’ depression status on young infants’ neurobehavioral profiles. Additionally, we should note that father’s depression status was ascertained through a third party informant report (by mothers). However, a validity study by Milne et al. showed that females were more likely than males to report with greater accuracy on the FHS.

Second, infant temperament and behaviors were assessed by maternal report using IBQ-R [22], and therefore, it is possible that mother’s depression status could have introduced a potential bias due to mothers’ perceptual inaccuracy when evaluating their infants’ temperament [33,34]. However, the use of IBQ-R has allowed us to quickly gain knowledge about infant temperament at an early age, which also may help serve as indicators in predicting their dispositions, instead of having to wait until the children are older. Furthermore, mothers may be the best informant for young infants. Nevertheless, more objective measures of infants’ temperament, for example, a third party report and/or behavioral observation would have improved the reliability and strengthened the findings. Thus, we have randomly selected 20 participants and obtained the temperamental profiles at one year in order to rectify our limitation, as temperament is considered to be relatively stable [35], using the information as indicators of test-retest reliabilities. Results show similar correlation among the 14 sub-scales (r ranging from 0.50 to 0.74). This limitation could have been somewhat rectified if the study had a longer follow-up. If parallel measures were used at 12 and 18 months instead of a partial sample, more meaningful insights could have been provided. The study will benefit greatly from a future follow-up study. Third, although we selected potential confounders, such as mothers’ race, marital status, and sex of the baby, which have been known as their associations with both predictors (i.e., depression) and outcomes (i.e., infant temperament), in our analyses as potential confounders, our sample size restricted us from considering a wider range of potential confounders. Even so, other confounders such as mothers’ socio-economic status, insurance status, alcohol and drug abuse, and other various demographics [2,3] may be useful to future studies to evaluate the association between parental depression (mothers and fathers) and infant and children behavior outcomes. Lastly, the current study lacks biological markers of the Hypothalamic-Pituitary-Adrenal (HPA) axis functioning, such as cortisol levels, which are known to be associated with depression [36]. Having a biological measure of maternal depression during pregnancy might help us further understand whether maternal and paternal depression influence infant temperament development differently, since infants (fetuses) can be directly exposed to maternal but not paternal cortisol levels.

Despite these limitations, the current study suggests that maternal well-being (i.e., lack of depression) during pregnancy has important consequences for child development, and that paternal well-being also plays an important role especially for their depressed partners (mothers). Future research should evaluate if treatment of paternal depression has an impact on maternal depression, which can improve mental health and family functioning overall. It is important for obstetricians, nurses, and midwives to actively play a role in assessing and identifying maternal depression during pregnancy and make this a routine practice. In addition, screening paternal depression, especially when mothers are depressed, may be an effective way of identifying at risk families. Increased awareness of the adverse effects of depression in pregnancy is necessary for future mothers and fathers, in order to improve well-being during pregnancy, and facilitate the most optimal environment, and subsequent birth outcomes for their unborn children.

## Conclusions

Infants of depressed mothers were directly associated with more reactive infant temperament on various IBQ-R items. Paternal depression during his partner’s pregnancy was not directly associated with more reactive infant temperament, but the interaction between maternal and paternal depression was an important predictor for infants’ reactive temperaments.

## Figures and Tables

**Table 1:** Demographic characteristics of mothers and fathers.

Demographic Characteristics	N (%)

Mother’s ethnicity	
Black	44 (33%)
Latina	67 (50%)
White	10 (7%)
Asian	8 (6%)
Other	6 (4%)

Mother’s educational attainment at delivery	
Primary school education	4 (3%)
Some high school/drop-out	40 (30%)
High school graduate or GED	28 (21%)
Some college	37 (28%)
College graduate	16 (12%)
Graduate degree	8 (6%)

Mother’s marital status at delivery	
Married/Common Law	32 (24%)
Single	97 (72%)
Divorced/Separated	6 (4%)

Welfare Status	
Private Insurance	16 (12%)
Medicaid	119 (88%)

Sex of baby	
Male	78 (56%)
Female	62 (44%)

	M (SD) range

Mother’s age (years)	27.3 (5.8) 16 – 43
Father’s age (years)	27.2 (6.9) 17 – 49

NB: N may vary due to missing values

**Table 2:** Main effect of maternal and paternal depression on infant temperament.

	Maternal Depression				Paternal Depression			
Temperament subscales	Not Depressed Mean (SD)	Depressed Mean (SD)	Statistics		Not Depressed Mean (SD)	Depressed Mean (SD)	Statistics	
			Unadjusted	Adjusted			Unadjusted	Adjusted
Act	4.38 (.17)	3.84 (.29)	F= 1.4, P= .24	F= .05, P= .83	**4.13 (.12)**	4.09 (.32)	F= .20, P= .65	F= 1.4, P= .23
Distress	3.54 (.18)	3.79 (.30)	F= 1.7, P= .20	F= 2.0, P= .16	3.80 (.12)	3.53 (.33)	F= .59, P= .44	F= .28, P= .60
Fear	3.50 (.19)	3.47 (.32)	F= .001, P= .98	F= .43, P= .52	3.44 (.13)	3.52 (.34)	F= .08, P= .77	F= .51, P= .48
Duration	4.96 (.20)	4.31 (.33)	F= 2.4, P= .12	F= 2.0 P= .16	4.84 (.14)	4.43 (.36)	F= .54, P= .47	F= .43, P= .51
Smiling & Laughter	6.15 (.16)	4.76 (.26)	**F= 7.8, P= .006**	**F= 4.8, P= .03**	5.71 (.11)	5.21 (.28)	F= .001, P= .99	F= .19, P= .66
High Pleasure	6.31 (.14)	5.18 (.23)	**F= 4.9, P= .03**	F= 2.2, P= .14	6.16 (.10)	5.33 (.26)	F= 2.5, P= .12	F= 1.1, P= .29
Low Pleasure	5.83 (.13)	5.40 (.22)	F= 2.5, P= .11	F= 3.0, P= .08	5.78 (.09)	5.45 (.24)	F= 1.0, P= .31	F= 1.3, P= .26
Soothability	5.85 (.14)	5.09 (.23)	**F= 5.4, P= .02**	**F= 4.5, P= .04**	5.50 (.09)	5.44 (.25)	F= .42, P= .52	F= .57, P= .45
Falling Reactivity	5.38 (.15)	4.80 (.24)	**F= 4.2, P= .04**	F= 2.9, P= .09	5.22 (.10)	4.96 (.27)	F= .28, P= .60	F= .14, P= .71
Cuddliness	6.08 (.14)	5.51 (.23)	F= 2.0, P= .16	F= 3.4, P= .07	5.83 (.09)	5.76 (.25)	F= .31, P= .58	F= .10, P= .75
Percept	4.60 (.24)	3.64 (.40)	F= 2.8, P= .10	F= .64, P= .42	4.50 (.16)	3.73 (.43)	F= 1.4, P= .24	F= .58, P= .45
Sadness	2.99 (.17)	3.69 (.28)	**F= 4.3, P= .04**	F= 3.4, P= .07	3.28 (.11)	3.41 (.30)	F= .01, P= .92	F= .005, P= .95
Approach	5.60 (.19)	4.56 (.31)	F= 2.9, P= .09	F= 1.7, P= .20	5.35 (.13)	4.77 (.34)	F= .44, P= .51	F= .26, P= .61
Vocal Reactivity	5.87 (.14)	4.39 (.22)	**F= 12.2, P= .001**	**F= 9.4, P= .003**	5.57 (.09)	4.70 (.24)	F= 2.0, P= .16	F= 1.08, P= .30

NB: In adjusted model, sex, race and marital status were statistically controlled for
